# Hypoxia Exacerbates Negative Emotional State during Inactivity: The Effect of 21 Days Hypoxic Bed Rest and Confinement

**DOI:** 10.3389/fphys.2018.00026

**Published:** 2018-02-08

**Authors:** Nektarios A. M. Stavrou, Tadej Debevec, Ola Eiken, Igor B. Mekjavic

**Affiliations:** ^1^School of Physical Education and Sport Science, National and Kapodistrian University of Athens, Athens, Greece; ^2^Sport Psychology Department, Hellenic Sports Research Institute, Olympic Athletic Center of Athens “Spyros Louis”, Athens, Greece; ^3^Department of Automation, Biocybernetics and Robotics, Jozef Stefan Institute, Ljubljana, Slovenia; ^4^Faculty of Sport, University of Ljubljana, Ljubljana, Slovenia; ^5^Department of Environmental Physiology, Swedish Aerospace Physiology Centre, Royal Institute of Technology, Stockholm, Sweden; ^6^Department of Biomedical Physiology and Kinesiology, Simon Fraser University, Burnaby, BC, Canada

**Keywords:** psychological responses, positive affect, negative affect, hypoxia, bed rest, simulated microgravity

## Abstract

Hypoxia and confinement have both been shown to influence emotional state. It is envisaged that the inhabitants of future planetary habitats will be exposed to concomitant confinement, reduced gravity and hypoxia. We examined the independent and combined effects of a 21-day inactivity/unloading and normobaric hypoxia under confined conditions on various psychological factors. Eleven healthy men participated in three 21-day experimental campaigns designed in a cross-over manner: (1) Normobaric hypoxic ambulatory confinement, (2) Normobaric hypoxic bed rest and (3) Normobaric normoxic bed rest. The Profile of Mood States, and the Positive and Negative Affect Schedule were employed to assess the participants' psychological responses before (Pre), during (Day 7, Day 14, and Day 21) and after (Post) the confinements. The most negative psychological profile appeared on days 14 and 21 of the hypoxic bed rest campaign. A significant increase in depression, tension, and confusion was noted on days 14 and 21 of the hypoxic bed rest condition. Concomitantly, a decrease, albeit not statistically significant, in positive psychological responses was observed. The psychological profile returned to the initial level at Post following all confinements. These data suggest that the combined effect of hypoxia and bed rest induced the most negative effects on an individual's mood. However, significant intra- and inter-individual differences in psychological responses were noted and should be taken into consideration.

## Introduction

Research in aid of human Space exploration has to date focused primarily on the microgravity-induced consequences of relative inactivity and unloading of the weight-bearing limbs, on integrative physiological responses. These studies have been performed during missions on the International Space Station (ISS), or by using ground-based simulations of microgravity-induced inactivity/unloading, such as the sustained bed rest (BR) model. While the BR studies to-date have focused extensively on investigating the effect of unloading/inactivity on physiological systems (Pavy-Le Traon et al., 2007), the analyses of the effects on psychological and emotional responses have been limited (Ishizaki et al., 2002; Liu et al., 2012). Nevertheless, both reduced gravity (Kanas et al., 2009) and unloading (Lipnicki and Gunga, 2009) have been shown to importantly modulate individuals' psychological responses. For logistical reasons, the environment within future habitats on the Moon and Mars will be hypoxic. Thus, in addition to confinement and reduced gravity (Eiken and Mekjavic, 2015), astronauts/cosmonauts will be exposed to continuous systemic hypoxia within the envisaged habitats. Given the fact that, similar to inactivity/unloading, hypoxia has also been shown to influence psychological status (Hornbein et al., 1989; Virués-Ortega et al., 2004), understanding the independent and combined effects of these factors on psychological indices is crucial for the success and safety of future space exploration.

The first and only study to-date (Stavrou et al., 2015) that investigated the combined effects of inactivity and hypoxia on psychological indices incorporated a cross-over repeated measures design, such that participants took part in three short-term interventions including normoxic BR (NBR), hypoxic BR (HBR), and hypoxic ambulation (HAMB). We (Stavrou et al., 2015) showed that 10-day NBR and HAMB interventions did not importantly influence the participants' psychological status. Conversely, HBR resulted in significant alterations, reflected in an increase of negative psychological responses such as negative affect, tension, anger, and confusion, and decrease of positive factors such as vigor, and positive affect. Based on Stavrou et al. (2015) results, it seems that exercise has a positive impact on psychological responses, increasing the positive and ameliorating the negative emotions. Previous research (Yeung, 1996; Hillman et al., 2008; Erickson et al., 2015) supports the positive impact of exercise on mood, affect, cognition, and brain activity, although these effects are highly dependent on exercise type/intensity as well as the participants' characteristics (e.g., age, physical condition).

To date, several studies have investigated the effects of altitude exposure on the indices of psychological status (Gallagher and Hackett, 2004; Bärtsch and Saltin, 2008). Reduction of O_2_ availability within the central nervous system, secondary to high altitude exposure induces a variety of neuropsychological impairments (Winget and DeRoshia, 1986; Hornbein, 2001) including alterations in cognition, mood, behavior and sleep indices. Other symptoms that are manifest during hypoxic exposure include impairments of coordination, vision, cognitive function, alertness, vigor, etc. (Acevedo and Ekkekakis, 2001; Virués-Ortega et al., 2004). Importantly, these symptoms are commonly complemented by increases of negative psychological and emotional responses and a decrease of the positive psychological profile elements (Yeung, 1996; Ishizaki et al., 2002; Lipnicki and Gunga, 2009). It has to be noted, however, that significant inter-individual differences in neuropsychological changes at high altitude have been reported (Hornbein et al., 1989; Hornbein, 2001). Indeed, large inter-individual differences might lead to different cognitive responses to various situations and consequently modulate an individuals' ability to adapt to adverse environments (Bahrke and Shukitt-Hale, 1993; Acevedo and Ekkekakis, 2001). Moreover, a moderating role of personality characteristics has also been suggested to influence the measured outcomes (Virués-Ortega et al., 2004).

Accordingly, the present study sought to determine the independent and combined effects of prolonged inactivity and hypoxia on select psychological indices and thereby extend our previous findings (Stavrou et al., 2015) obtained during a shorter exposure time (10 days). This is especially important given that the up-to-date body of research on psychological responses to hypoxic or bed rest environments is limited, and moreover the prolonged combined effect of both factors has not yet been investigated. The results of the current study do not only have application for space missions and performance settings but also have an important clinical aspect for many patients rendered both hypoxic and inactive as a consequence of cardiovascular of respiratory diseases.

## Methods

This study was a sub-project of larger international research investigation into the physiological and psychological effects of simulated planetary habitation on healthy humans. All experimental procedures within the project were conducted according to the European Space Agency (ESA) bed rest standardization recommendations (Standardization of bed rest study conditions 1.5, August 2009). These recommendations have been developed to standardize the methodology of bed rest studies. This allows the comparison of results from studies conducted at different facilities. The outlined recommendations ensure that the main aspects of each study, particularly those related to overall study protocol, participants selection, medical care and data acquisition are uniform to the highest degree possible. The study was approved by the National Medical Ethics Committee of the Republic of Slovenia, registered at ClinicalTrials.gov (NCT02293772) and conducted according to the Declaration of Helsinki guidelines. The current study focused on psychological and emotional responses before, during and after the experimental interventions.

### Participants

Besides the general inclusion/exclusion criteria outlined in the protocol, individuals recently (<2 months) exposed to altitudes above 2,000 m were ineligible to participate. Following comprehensive interviews, fitness tests, and medical examinations, 14 participants were invited to participate in the study. They were provided with an information booklet detailing all experimental protocols, and were also given oral presentations regarding the experimental protocols included in the overall study. All participants gave their written informed consent prior to inclusion in the study. Two participants did not complete the last campaign while one participant had to be withdrawn from the study during the course of the last campaign due to a medical issue that was not related to participation in the study. Of the original 14 participants who entered the study, eleven healthy, sea level residents (age: 27 ± 6 years (mean ± SD); stature: 180 ± 3 cm; body mass: 77 ± 12 kg; BMI: 23.7 ± 3.0 kg·m^−2^; body fat %: 21 ± 5%; VO_2max_: 44.3 ± 6.1 mL·kg^−1^·min^−1^) completed all trials and thus only their data is included in the present analysis.

### Study outline

The project outline has been extensively described previously (Debevec et al., 2014). Briefly, the participants underwent the following three, separate campaigns in a randomized, counterbalanced manner: (1) normobaric normoxic bed rest (NBR; partial pressure of inspired oxygen, P_i_O_2_ = 133.1 ± 0.3), (2) normobaric hypoxic ambulatory confinement (HAMB; P_i_O_2_ = 90.0 ± 0.4; ~4,000 m simulated altitude), and (3) normobaric hypoxic bed rest (HBR; P_i_O_2_ = 90.0 ± 0.4; ~4,000 m simulated altitude). All experimental interventions were performed at the Olympic Sport Centre Planica (Rateče, Slovenia), located at 940 m above sea level. Two participants per day entered each campaign in a sequential and fixed order. For participants, each of the three campaigns lasted 32 days and comprised the following three phases: (1) The initial testing phase (Pre) lasted 7 days. This phase enabled the participants to familiarize themselves with the experimental facility, and the researchers to obtain the baseline measures (Pre). (2) The confinement phase lasted 21 days (Day 1–Day 21), during which each participant was assigned to their designated environmental condition (NBR, HAMB, & HBR). (3) A four-day recovery phase that enabled cautious re-ambulation of the participants and obtainment of the post-intervention measurements (Post). A 4-month recovery period was implemented between each campaign to enable sufficient wash-out of physiological and psychological intervention effects.

### Bed rest and hypoxic standard procedures

Throughout all three study campaigns, the participants were accommodated in double bed-rooms with two participants accommodated in each room. The lights were turned on and the participants awakened at 7:00 a.m. daily and room lights were turned off at 11:00 p.m. Napping was not allowed during the day hours. The facility environmental conditions were constantly controlled and remained stable during the three campaigns (ambient temperature = 24.4 ± 0.7°C; relative humidity = 53.5 ± 5.4% and ambient pressure = 684 ± 4 mm Hg). During the experimental campaigns, participants were able to communicate and socialize with other participants and/or research personnel either in their rooms or within the joint common area of the facility. In the latter case, participants were transported to the common area by a gurney, and maintained a horizontal position on the mattresses provided in the common area. Also, high-speed internet connection and a personal tablet computer were provided to each participant. No targeted cognitive tasks or mental stimulations were applied.

The participants were confined to strict horizontal BR during the NBR and HBR campaigns. Horizontal BR has been identified as a valid ground-based model to simulate microgravity-induced physiological adaptations (Pavy-Le Traon et al., 2007). All habitual activities (e.g., showering, lavatory) were performed in the supine position. For head support, the participants were allowed to employ one head-pillow. No physical activity, besides changing recumbent position, was allowed during the confinements. Compliance to the BR protocol was monitored using continuous CCTV and permanent medical supervision. To alleviate potential backache during the initial days of each BR the participants were provided with passive physiotherapy.

During HAMB the participants were encouraged to move freely in the common hypoxic area (110 m^2^ surface) and encouraged to engage in their usual daily routines. The HAMB participants also performed two, 30-min exercise sessions of low intensity each day to mimic their normal habitual activity levels. The exercise mode (stepping, dancing or cycling) was rotated daily.

The normobaric hypoxic environment within the facility was generated by the Vacuum Pressure Swing Adsorption system (b-Cat, Tiel, The Netherlands) that supplied the O_2_-depleted gas to the designated rooms and the common hypoxic area. The ambient air within the rooms was monitored and analyzed for O_2_ and CO_2_ content at regular intervals (15-min). In the event of a variation in the O_2_ levels the system restored the target O_2_ levels immediately. During all hypoxic campaigns, the participants wore, or had in close proximity, a portable ambient O_2_ concentration analysers (Rae PGM-1100, California, USA) that activated a safety audible alarm, if the O_2_ level decreased below the pre-set level.

## Experimental procedures

### Daily physiological monitoring

In order to monitor the participants general adaptation during the campaigns, heart rate (HR), capillary oxygen saturation (SpO_2_) and arterial pressures (AP) were measured each morning upon awakening using a HR telemetry device (iBody, Wahoo Fitness, Atlanta, USA), finger oximetry device (3100 WristOx, Nonin Medicals, Minnesota, USA) and manual sphygmomanometer (Diplomat-presameter, Riester, Jungingen, Germany), respectively.

### Acute mountain sickness assessment

To assess the presence and severity, of acute mountain sickness (AMS) during the confinement the participants were requested to complete the Lake Louise AMS questionnaire daily (Roach et al., 1993). The Lake Louise score (LLS; 0-15) was consequently calculated and the score of ≥3 along with a presence of headache was used to define AMS occurrence.

### Profile of mood states

The participants' mood state was assessed using the Profile of Mood States - Short Form (POMS-SF) (Shacham, 1983). The POMS-SF is a 37-item self-evaluation questionnaire comprised the following six subscales: tension-anxiety, depression-dejection, anger-hostility, vigor-activity, fatigue-inertia, and confusion-bewilderment. The participants' provided their subjective mood states on a 5-point scale ranging from 0 “not at all” to 4 “extremely.” Several validation studies (Curran et al., 1995) lent support for the internal consistency and test-retest reliability of the POMS-SF subscales. The reliability of the POMS subscales (Cronbach *a*; Cronbach, 1951) in the current study was acceptable (tension = 0.67–0.88, depression = 0.76–0.95, vigor = 0.81–0.91, anger = 0.79–0.92, fatigue = 0.79–0.94, confusion = 0.79–0.92).

### Positive and negative affect schedule

The Positive and Negative Affect Schedule (PANAS) (Watson et al., 1988) is also a self-report instrument comprising 20 items. It was initially developed to enable the measurement of respondents' positive affect (PA; 10 individual items) and negative affect (NA; 10 individual items). The participants were asked to rate the extent to which they experienced each of the noted affects outlined in PANAS. Participants provided their answers on a 5-point scale with anchors between “*very slightly or not at all*” (1) to “*extremely*” (5). The PANAS indicated acceptable reliability in the current study (Cronbach *a*: negative affect = 0.91–0.95, positive affect = 0.78–0.96).

The participants completed both of these psychological instruments (POMS &PANAS) before, during and after each confinement period. In particular, the data were obtained two days before the onset of intervention (PRE), on the 7th (D7), 14th (D14), and 21st (D21) days of each intervention and 1 day following the intervention cessation (POST). At each time point, the participants were instructed to complete the questionnaires solely based on their feelings exactly at the time the questionnaire was administered.

### Statistical analysis

A multivariate analysis of variance (Conditions: NBR, HBR, HAMB) × 5 (Time: PRE, D7, D14, D21, POST), with repeated measures on the second factor (RMANOVA) were performed for subscales of both PANAS and POMS. In addition, separate analyses of variance were, based on the RMANOVA outcomes, performed on each of the affect (PANAS) and mood (POMS) factors to determine the between-participant differences in each experimental condition and for the within-participant repeated measures within the conditions. When the assumptions of sphericity were not met in the within-participants repeated measure analyses, the Green-House Geisser correction and the corresponding degrees of freedom, were used to estimate the *F* statistic (Tabachnick and Fidell, 2006; Field, 2009). A *post hoc*- Bonferroni corrected *t*-tests were employed to determine any significant differences between the pairwise ANOVA comparisons (Tabachnick and Fidell, 2006; Field, 2009). In order to determine the relationship between the measured physiological and psychological indices a pooled Pearson's correlation analysis was employed. The significance level was set *a priori* at 0.05. The Statistical Package for Social Sciences (SPSS 21.0 Win) was employed for all of the data and statistical analyses.

## Results

### General adaptation

With the exception of transient head- and back-aches the participants did not experience any significant adverse health-related issues. Due to severe hypoxemia (SpO_2_ < 75%) and dizziness of one participant during the initial exposure to HBR intervention, he was individually exposed to a simulated altitude of ~3,000 m and ~3,500 during the next 2 days. He recommenced his exposure to 4,000 m on Day 3 without any additional adverse effects. The same protocol was thereafter replicated during the HAMB campaign. As noted in Table 1 the resting HR was significantly higher during the HAMB and HBR compared to NBR (*p* < 0.05), but remained stable throughout each confinement period. As expected, during both hypoxic confinements (HAMB, HBR) SpO_2_ was significantly reduced (*p* < 0.001) compared to PRE values and values observed during NBR. Both, systolic and diastolic AP values were significantly higher during the HAMB and HBR compared to the NBR campaign (*p* < 0.01). Compared to PRE values, AP was significantly higher on D7 and D21 during the HAMB and D14 and D21 during the HBR. As reported previously (Debevec et al., 2014), the subjective AMS symptoms were noted in three participants during the HAMB and five participants during the HBR campaign on the first 2 days of the confinements. Moreover, significantly higher values of LLS were observed during the D7 and D21 of the HAMB and HBR campaigns as compared to NBR.

**Table 1:** Means *(M)* and Standard Deviations *(SD)* of the Heart Rate (HR), Capillary Oxyhemoglobin Saturation (SpO_2_), Arterial Pressure (AP), and Lake Louise Score (LLS) values before during and after experimental conditions.

**Campaign**	**Day**	**HR**	**SpO_2_**	**AP (mmHg)**		**LLS**
		**(beats·min^−1^) *M* (*SD*)**	**(%) *M* (*SD*)**	**Systolic *M* (*SD*)**	**Diastolic *M* (*SD*)**	**a.u. *M* (*SD*)**
HAMB	PRE	64 (9)	97 (1)	111 (12)	65 (4)	0.8 (0.9)
	D7	66 (9)^#^	87 (3)^*^^#^	122 (9)^*^^#^	75 (8)^*^^#^	0.9 (1.8)^#^
	D14	67 (10)^#^	88 (2)^*^^#^	115 (11)^#^	70 (9)^#^	0.7 (1.2)
	D21	67 (8)	89 (3)^*^^#^	116 (16)^#^	74 (8)^*^^#^	1.1 (1.8)^#^
	POST	65 (12)	97 (1)	111 (12)	68 (9)	0.8 (1.5)
HBR	PRE	63 (11)^#^	97 (1)	114 (11)	64 (6)	0.2 (0.4)
	D7	72 (10)^*^^#^	88 (2)^*^^#^	118 (10)^#^	70 (8)^#^	0.9 (1.2)^#^
	D14	75 (7)^*^^#^	89 (1)^*^^#^	118 (10)^#^	75 (10)^*^^#^	1.2 (1.6)
	D21	74 (13)^*^^#^	90 (2)^*^^#^	119 (11)^*^^#^	74 (10)^*^^#^	1.1 (1.1)^#^
	POST	68 (9)	97 (1)	115 (13)	65 (12)	2.5 (1.9)^#^
NBR	PRE	63 (11)	98 (1)	108 (12)	62 (5)	0.7 (1.2)
	D7	57 (6)^*^	97 (1)	108 (10)	65 (8)	0.1 (0.3)^*^
	D14	59 (10)	97 (1)	109 (11)	68 (7)^*^	0.9 (1.8)
	D21	63 (11)	97 (1)	109 (10)	67 (8)	0.4 (0.9)
	POST	66 (8)	98 (1)	105 (7)	64 (5)	1.3 (2.2)

PRE, 2 days before the onset; D7, 7th day; D14, 14th day; D21, 21st day; POST, first day after; HAMB, hypoxic ambulatory condition; HBR, hypoxic bed rest condition; NBR, normoxic bed rest condition;

* Denotes significant difference compared to PRE (p < 0.05);

# *Denotes significant difference compared to corresponding NBR values (p < 0.05)*.

### Baseline psychological responses

The baseline responses to the PANAS (PA: positive affect, NA: negative affect) and POMS subscales (tension, vigor, anger, fatigue, confusion, and depression) in the three experimental conditions (HAMB, HBR, and NBR) were obtained in the pre-intervention period, 2 days before the start of each 21-days intervention. No significant baseline differences were noted on either the PANAS (*F* = 0.49, *ns*, ${\text{η}}_{\text{p}}^{2}$ = 0.03), or the POMS (*F* = 0.84, *ns*, ${\text{η}}_{\text{p}}^{2}$ = 0.16) subscales between the experimental conditions.

### Positive and negative affect schedule

**Figure 1:**
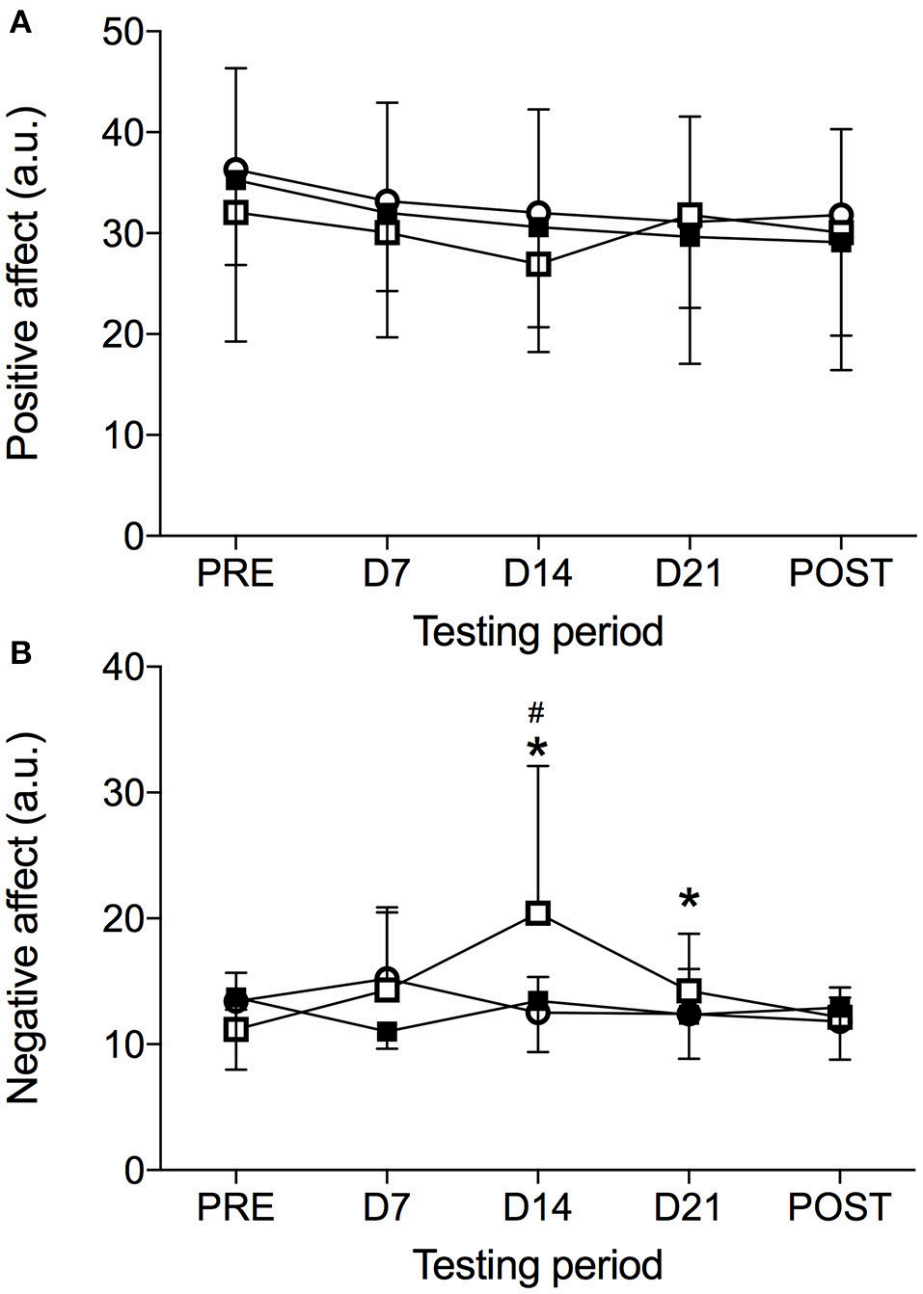
The Positive Affect and Negative Affect Schedule values [PANAS, Positive Affect **(A)** and Negative Affect **(B)**] obtained before (PRE), on days 7 (D7), 14 (D14), 21 (D21), and immediately after (POST) hypoxic ambulatory condition (HAMB, open circles), hypoxic bed rest condition (HBR, open squares) and normoxic bed rest (NBR, full squares) condition. *Denotes significant difference within the HBR condition compared to PRE (*p* < 0.05); ^#^Denotes significant difference within the HBR condition compared to POST (*p* < 0.05).

The data from the PANAS subscales in each condition is presented in Figure 1. Significant Condition × Time interaction was noted (*F* = 1.91, *p* < 0.05, ${\text{η}}_{\text{p}}^{2}$ = 0.11) for the PANAS subscales. The means and standard deviations of the PANAS subscales are presented in Figure 1. The results indicate that there was a significant Condition X Time interaction for NA (*F* = 3.36, *p* < 0.01, ${\text{η}}_{\text{p}}^{2}$ = 0.18). A significant difference for the NA was observed in the HBR condition (*F* = 4.24, *p* < 0.05, ${\text{η}}_{\text{p}}^{2}$ = 0.28), demonstrating an increase in NA from PRE to D14 (*p* < 0.05) and D21 (*p* < 0.05). NA later decreased from D14 to POST (*p* < 0.05). No significant changes over Time were noted during NBR (*F* = 1.30, *p* = 0.294, ${\text{η}}_{\text{p}}^{2}$ = 0.12) or the HAMB conditions (*F* = 2.42, *p* = 0.104, ${\text{η}}_{\text{p}}^{2}$ = 0.21). The results do not indicate a significant Condition × Time interaction for PA (*F* = 0.57, *p* = 0.799, ${\text{η}}_{\text{p}}^{2}$ = 0.04; Figure 1).

### Profile of mood states

The results of the POMS subscales are detailed in Table 2. An observed significant interaction for the POMS subscales (Condition × Time) (*F* = 2.08, *p* < 0.001, ${\text{η}}_{\text{p}}^{2}$ = 0.12), indicates that mood-state was changing over time differently during the three experimental conditions.

**Table 2:** Descriptive Statistics of the Profile of Mood State (POMS) subscale values before during and after experimental conditions.

**POMS subscales**	**Condition**	**PRE *M* (*SD*)**	**D7 *M* (*SD*)**	**D14 *M* (*SD*)**	**D21 *M* (*SD*)**	**POST *M* (*SD*)**
Tension	HAMB	2.36 (2.50)	3.36 (2.91)	3.27 (4.31)	3.64 (4.54)	1.73 (2.15)
	HBR	2.25 (1.86)	4.92 (4.66)	8.33 (5.66)	7.58 (4.12)	3.92 (2.53)
	NBR	2.00 (2.83)	1.64 (1.96)	5.36 (3.47)	3.18 (3.34)	1.45 (1.97)
Vigor	HAMB	12.54 (6.01)	10.73 (6.39)	10.82 (5.19)	12.36 (4.63)	11.73 (5.23)
	HBR	9.91 (5.97)	10.17 (5.27)	9.25 (4.33)	10.08 (5.50)	9.58 (4.98)
	NBR	12.82 (6.34)	11.36 (5.71)	10.73 (6.53)	8.82 (6.24)	9.82 (5.10)
Anger	HAMB	0.72 (1.19)	3.09 (4.59)	4.18 (5.83)	3.27 (4.96)	1.55 (2.66)
	HBR	1.92 (2.47)	3.50 (4.91)	2.50 (3.90)	2.50 (2.50)	1.58 (1.73)
	NBR	1.55 (3.39)	1.18 (1.72)	2.55 (3.27)	2.82 (5.36)	1.36 (2.20)
Fatigue	HAMB	2.73 (3.55)	2.73 (3.50)	4.82 (5.88)	2.82 (2.86)	2.55 (2.34)
	HBR	2.75 (2.53)	2.33 (2.96)	2.25 (3.47)	2.58 (2.07)	6.58 (4.58)
	NBR	2.91 (3.86)	1.55 (2.12)	2.73 (3.50)	1.73 (2.33)	4.18 (3.82)
Confusion	HAMB	1.00 (2.05)	2.45 (3.96)	3.36 (5.35)	1.36 (2.29)	1.00 (1.79)
	HBR	0.75 (1.14)	2.50 (2.81)	4.92 (4.40)	4.75 (2.96)	2.50 (2.58)
	NBR	1.73 (3.47)	1.18 (2.40)	2.55 (3.91)	1.45 (2.16)	1.64 (3.04)
Depression	HAMB	0.73 (1.42)	4.64 (4.03)	3.27 (4.69)	1.82 (3.79)	1.09 (2.21)
	HBR	0.50 (1.73)	4.50 (6.20)	4.42 (4.72)	6.42 (3.96)	2.08 (1.73)
	NBR	1.45 (4.50)	2.27 (2.80)	3.73 (3.55)	2.36 (1.50)	1.64 (2.25)
TMD	HAMB	19.00 (12.30)	29.55 (21.97)	32.09 (27.94)	24.55 (18.12)	20.18 (13.88)
	HBR	22.25 (12.69)	31.58 (22.98)	37.17 (20.74)	37.75 (15.98)	31.08 (13.63)
	NBR	20.82 (17.85)	20.45 (11.24)	30.18 (19.76)	26.73 (16.85)	24.45 (12.86)

*PRE, 2 days before the onset; D7, 7th day; D14, 14th day; D21, 21st day; POST, first day after; HAMB, hypoxic ambulatory condition; HBR, hypoxic bed rest condition; NBR, normoxic bed rest condition; TMD, total mood disturbance*.

The analysis of depression showed a significant Condition × Time interaction (*F* = 2.36, *p* < 0.05, ${\text{η}}_{\text{p}}^{2}$ = 0.13). The depression values in HAMB (*F* = 4.30, *p* < 0.01, ${\text{η}}_{\text{p}}^{2}$ = 0.30) changed significantly over time. Depression on D7 was higher than PRE (*p* < 0.05) and D21 (*p* < 0.01) measures. Compared to D7, the POST values also approached statistical significance (*p* = 0.057). Additionally, differences were revealed during HBR (*F* = 6.70, *p* < 0.01, ${\text{η}}_{\text{p}}^{2}$ = 0.38), showing an increase of depression in the D21 measures compared to PRE (*p* < 0.01) and POST (*p* < 0.05) measures. Also, the difference between D21 and D14 approached statistical significance (*p* = 0.059). Finally, no significant depression-level changes appeared over time in NBR trial (*F* = 1.50, *p* = 0.248, ${\text{η}}_{\text{p}}^{2}$ = 0.13). When we examined the differences between the three conditions, the differences were only significant on D21, with HBR demonstrating higher depression levels compared to both, the NBR (*p* < 0.05) and HAMB (*p* < 0.01).

A significant Condition × Time interaction for fatigue (*F* = 2.36, *p* < 0.05, ${\text{η}}_{\text{p}}^{2}$ = 0.13) was detected. The results indicate that fatigue significantly changed in the HBR condition (*F* = 5.45, *p* < 0.05, ${\text{η}}_{\text{p}}^{2}$ = 0.33). There were no significant fatigue differences across time during hypoxic BR exposure, except for the difference between D21 to POST, which approached statistical significance (*p* = 0.06). No significant time-related changes were observed in both, the HAMB (*F* = 1.19, *p* = 0.318, ${\text{η}}_{\text{p}}^{2}$ = 0.11), and the NBR condition (*F* = 2.01, *p* = 0.158, ${\text{η}}_{\text{p}}^{2}$ = 0.17). Examining the difference between the experimental conditions across the five time measures, significant differences were only noted on the POST measure (*F* = 3.42, *p* < 0.05), during which the HBR condition showed higher fatigue than the HAMB condition (*p* < 0.05).

The results for the tension subscale indicated a significant Condition × Time interaction (*F* = 2.75, *p* < 0.001, ${\text{η}}_{\text{p}}^{2}$ = 0.15). A significant fluctuation in tension over time was noted in the HBR (*F* = 10.90, *p* < 0.001, ${\text{η}}_{\text{p}}^{2}$ = 0.50). Specifically, tension was augmented on D14 (*p* < 0.05) and on D21 (*p* < 0.01) compared to PRE and then reduced on POST measure compared to D14 (*p* < 0.05) and D21 (*p* < 0.01). Differences were also revealed during NBR (*F* = 7.55, *p* < 0.01, ${\text{η}}_{\text{p}}^{2}$ = 0.43), showing that the D14 measure of tension increased compared to D7 (*p* < 0.05), while decreased on POST measure (*p* < 0.05). On D14 of the HBR condition tension was higher when compared to the HAMB (*p* < 0.05). Anger was higher in the HBR condition in the D21 and POST measures compared to the NBR (*p* < 0.05). For tension, no differences were noted across time in the HAMB (*F* = 1.11, *p* = 0.353, ${\text{η}}_{\text{p}}^{2}$ = 0.10).

A significant Condition × Time interaction was revealed for confusion (*F* = 2.77, *p* < 0.05, ${\text{η}}_{\text{p}}^{2}$ = 0.15). Significant changes were noted within the HBR condition (*F* = 9.26, *p* < 0.001, ${\text{η}}_{\text{p}}^{2}$ = 0.46). *Post hoc* analysis indicated that confusion increased on D14 (*p* < 0.05) and D21 (*p* < 0.001) compared to PRE measure. On the other hand, confusion decreased from D21 to POST (*p* < 0.05) during HBR. The between conditions analysis indicates that on D21 (*F* = 6.87, *p* < 0.01) significant differences were revealed between the HBR and NBR (*p* < 0.01) and HAMB conditions (*p* < 0.01). No significant Condition × Time interaction for NBR (*F* = 1.36, *p* = 0.280, ${\text{η}}_{\text{p}}^{2}$ = 0.12) or HAMB (*F* = 2.23, *p* = 0.147, ${\text{η}}_{\text{p}}^{2}$ = 0.18) was found.

Finally, no significant Condition × Time interaction was noted for vigor (*F* = 0.92, *p* = 0.482, ${\text{η}}_{\text{p}}^{2}$ = 0.06) and anger (*F* = 0.89, *p* = 0.502, ${\text{η}}_{\text{p}}^{2}$ = 0.05) was detected. Based on the POMS subscales, we subsequently calculated the total mood disturbance score [TMD = tension + depression + anger + confusion + fatigue + (24 – vigor)] (Curran et al., 1995) and compared it among the three experimental conditions. The results indicate no significant Condition X Time interaction (*F* = 1.11, *p* = 0.363, ${\text{η}}_{\text{p}}^{2}$ = 0.07) for TMD.

### Correlations between physiological and psychological indices

The only two physiological indices that were not significantly correlated to any of the reported psychological variables were Vigor and PA. On the other hand, the LLS values were only significantly correlated to the Fatigue subscale (*r* = 0.75, *p* < 0.001). A significant correlation with the changes in the SpO_2_ were noted for Tension (*r* = −0.55, *p* < 0.05) Anger (*r* = −0.80, *p* < 0.001), Confusion (*r* = −0.61, *p* < 0.05), Depression (*r* = −0.70, *p* < 0.001), TMD (*r* = −0.65, *p* < 0.001) and NA (*r* = −0.51, *p* < 0.05). Similarly, changes in HR in each condition were correlated to Tension (*r* = 0.70, *p* < 0.05), Confusion (*r* = 0.72, *p* < 0.01), Depression (*r* = 0.50, *p* < 0.05), TMD (*r* = 0.72, *p* < 0.001) and NA (*r* = 0.64, *p* < 0.01). Finally, changes in mean AP also indicated significant correlations to Tension (*r* = 0.65, *p* < 0.001), Anger (*r* = 0.57, *p* < 0.05), Confusion (*r* = 0.62, *p* < 0.01), Depression (*r* = 0.68, *p* < 0.05), TMD (*r* = 0.65, *p* < 0.01), and NA (*r* = 0.53, *p* < 0.05).

## Discussion

The main finding of the present study is that prolonged hypoxic inactivity significantly augmented negative mood. This also lends further support to the notion that hypoxia exacerbates negative emotions usually noted during BR of longer duration. Participants did not exhibit any such psychological changes during the HAMB condition, suggesting that even habitual levels of physical activity might counteract the negative effects of hypoxia on mood and emotional status. Finally, it is also noteworthy that significant variation in the intra- and inter-individual psychological responses was observed in both the hypoxic and normoxic BR condition.

### General adaptation

As noted in Table 1, significantly lower SpO_2_ values were noted during both hypoxic conditions. This reduction in SpO_2_ was a direct consequence of the lower ambient O_2_ in the HBR and HAMB as compared to the NBR condition, which also resulted in higher daily heart rate and arterial pressure levels. Based on the performed correlation analysis and given the strictly controlled environmental conditions in the present study the observed differences in the psychological status of subjects between the NBR and HBR conditions can most likely be attributed to systemic hypoxemia and other consequential physiological responses. It is noteworthy, that during HAMB participants were also continuously confined to the hypoxic facility, but did not exhibit any negative responses. This may be, in part, due to the fact that they spent time with the HBR participants in their rooms, which ensured more social interaction among the participants then commonly reported for previous BR studies. Undoubtedly, this social interaction attenuated the psychological effects of confinement *per se*.

### The effect of bed rest

In the NBR condition, a significant increase appeared in tension on D14 compared to the PRE measure, while a decrease was noted at POST, immediately following the BR condition. Although non-significant changes appeared in the remaining psychological factors it is important to note that depression and TMD increased on D14 and D21, and decreased only after the end of the BR exposure. On the other hand, non-significant changes were revealed in participants' positive psychological state (positive affect, vigor), which is in contrast to previous findings (Ishizaki et al., 2002; Liu et al., 2012; Stavrou et al., 2015). There are two key differences between the present study and our previous study (Stavrou et al., 2015): (1) the duration of the present study (21 days) was double the duration of the previous one (10 days), and (2) as noted above, much more emphasis was placed on the social interaction between participants, and between participants and staff. It is important to note that the BR-related increase in negative emotions can also be related to the confinement *per se*, as well as to the social isolation and subsequent lack of social interaction (Palinkas, 2001; Manzey, 2004; Kanas et al., 2007).

Based on the results of the current and previous work, it seems that the effect of BR on emotional responses and cognitive function is currently still unclear. Although most of the studies suggest detrimental effects of BR (Ryback et al., 1971; Edwards et al., 1981; Stavrou et al., 2015), others found either an improvement during BR (DeRoshia and Greenleaf, 1993; Ishizaki et al., 2002) or no changes (Shehab et al., 1998). Such equivocal findings might be due to duration of BR exposure or might be underlined by differences in participants' emotional characteristics, such as the manner in which they individually respond and adapt to adverse environments', and the management of negative emotions. It may be more appropriate to examine participants' individual psychological responses, rather than average or median results, as this approach would take into account the role of personality characteristics in the adaption to adverse environments. Future research should not only focus on psychological responses during bed rest and/or hypoxic exposure, but also examine participants' personality characteristics. This approach would be especially valuable during the process of selecting crew members for space missions. Individuals that have an appropriate personality profile, leadership and coping skills may have an advantage in adapting to space habitat environments ensuring a successful completion of a space mission (Palinkas, 2001). In addition, better understanding of the potential detrimental effects of extremely low levels of physical activity has important implications for clinical populations (Park et al., 2001; McDonald, 2002; Gaul et al., 2006).

### The effect of hypoxia

Our results indicate that the HBR intervention elicited the most negative emotional state compared to the NBR and HAMB conditions. HBR participants exhibited higher negative affect, depression, fatigue, and tension compared to the participants of the NBR and the HAMB conditions. In contrast, no significant effect was noted in positive emotions such as positive affect and vigor, which is in accordance with our previous findings (Stavrou et al., 2015).

The combination of BR and hypoxia exerts a detrimental effect on participants' mood, as reflected by the consecutive increase of depression and tension, remaining on a high level on D14 and D21. Participants in the HBR compared to those in the HAMB condition exhibited higher tension and depression on D14 and D21, respectively, indicating the protective role of exercise in the deterioration of mood in adverse environments. In addition to the above, it is important to note that the HAMB participants showed a significant increase of depression on D7 compared to PRE measure. However, the fact that depression levels decreased thereafter is suggestive of participants' successful adaptation to the respective conditions. It is also noteworthy that fatigue in the HBR condition reached the highest value in the POST measure. This increase in fatigue can be attributed to either participants' mood state during the HBR exposure, or it can be linked to personality characteristics as there was a large variability in participants' responses, ranging from 0 to 14.

The current results are in line with previous research findings (Banderet and Burse, 1991; Bahrke and Shukitt-Hale, 1993; Davidson, 2001; Stavrou et al., 2015) supporting the notion that terrestrial altitude exposure-related reduced systemic O_2_ supply to the nervous system can provoke significant negative effects on the neuropsychological state and adaptation. In addition, regarding the effect of altitude exposure on psychological responses, future investigations should examine the relation between an individual's cognitive function and related psychological responses in order to further elucidate the key factors that enable efficient adaptation to adverse environments.

In summary, based on the current and previous results (Stavrou et al., 2015) of the combined effect of bed rest and hypoxia on psychological indices, it seems that both factors significantly contribute to detrimental effect on mood, as hypoxia provokes negative psychological responses mainly in the inactive, bed ridden participants. Interestingly, such response is not evident in the hypoxic ambulatory participants, indicating that even low activity levels can be beneficial as has also been previously reported during protocols of short duration (Stavrou et al., 2015).

### The effect of activity

Comparison of the psychological responses between the HBR and HAMB clearly shows that the participants in the HBR condition revealed the most negative psychological profiles on D14 and D21, when their psychological profile continuously deteriorated. Specifically, the participants in the HBR compared to those of the HAMB condition showed a more pronounced negative mood, consisting of higher tension, depression, confusion, and fatigue. It seems that the regular daily physical activity as well as other daily activities performed by the participants in the HAMB condition significantly blunted the psychological impairment observed in the HBR condition.

Participants' mood state was consistently compromised during the HBR intervention, as demonstrated by the significant changes in most of the negative psychological factors observed during the hypoxic exposure as compared to the baseline. Conversely, the emotional responses remained fairly stable during the HAMB condition with no significant differences during the hypoxic exposure. This is indicated by the non-significant differences for positive or negative emotions during intervention (D7, D14, and D21) compared to PRE or POST measures. The only significant alteration for the HAMB participants was limited to an increase of depression on D7 compared to PRE measure. However, this may be a transient augmentation of depression as a consequence of hypoxic exposure, with values returning to the initial pre-intervention level as a result of the beneficial effect of exercise. This is further supported by recent research findings underlying exercise benefits for depression treatment (Barbour and Blumenthal, 2005; Russo-Neustadt and Chen, 2005).

The daily physical activity of the HAMB participants was meant to mimic participants' normal life daily activity, and was not meant to provide a training stimulus. This daily physical activity during HAMB seemed sufficient to ameliorate the deterioration of participants' psychological responses during HBR. Extensive research has supported the positive effect of exercise on psychological responses and cognitive function (Ekkekakis, 2003; Hillman et al., 2008; Erickson et al., 2015). However, the independent effects of various activity levels on psychological responses during hypoxic/altitude exposures have, to-date been largely ignored. Our results seem to be in line with previous studies supporting the positive effect of exercise on different psychological variables as well as on cognitive function (Hillman et al., 2008; De La Torre et al., 2012; Erickson et al., 2015). Yeung (1996) review on the effects of exercise on mood and mental health, clearly shows that (85%) of the studies report improved mood and, moreover, that this benefit seems to be at least partially dependent on the duration and intensity of the activity (Lind et al., 2005; Ekkekakis and Lind, 2006). Gordon et al. (2008), Hötting and Röder (2013), and Weinstein et al. (2012) support the important role of cardiorespiratory fitness and physical exercise in the facilitation of neuroplasticity and greater prefrontal cortex volume, and the improvement of cognitive functioning. Cognitive functioning seems to have a mediating role in participants' experience of negative emotions providing a better adaptation to stressful and adverse environments (Acevedo and Ekkekakis, 2001). It is however, noteworthy, that contradictory results appeared in BR studies regarding the effect of exercise on mood and emotion. In particular, some studies suggest that short-term exercise interventions are already sufficient to restore mood and brain cortical function to pre-isolation values (Van Baarsen et al., 2009), while others did not reveal any effect of exercise, since it did not limit the deterioration of psychological responses (DeRoshia and Greenleaf, 1993; Ishizaki et al., 2002).

### Implications for clinical populations

Patients with various clinical conditions (e.g., chronic obstructive pulmonary disease, heart failure), rendering them less-active also have systemic hypoxia-related elevated levels of negative psychological responses and reduced exercise capacity (Ritz et al., 2000; Mikkelsen et al., 2004). This exerts a detrimental effect on their quality of life, and establishes a vicious cycle of declining physical activity and increasingly unpleasant perception of exercise, affecting them both physically and emotionally (Troosters et al., 2013). Wilson (2006) revealed that increased levels of depression and anxiety can be noted in severe COPD patients, and moreover that the psychological effects can consequently contribute to COPD worsening. Based on the aforementioned results, the BR model combined with hypoxia might be a useful simulation providing fruitful information on the treatment of chronically ill patients who are rendered inactive and hypoxic. While, it is currently speculative whether implementing appropriate activity levels in this population would contribute to reduction of their negative psychological responses, it is certainly an area worthy of further consideration, having in mind the positive or protective effect of exercise in mood during hypoxia.

### Implications for planetary habitats

Bed rest studies are normally conducted to examine the effects of unloading/inactivity and not to mimic the exact activity of the astronauts in Space habitats. As such they cannot be considered a high-fidelity analog of a Space mission, and are also not designed for such simulations. Since the boundary conditions of future Lunar habitats are as yet unresolved, the present study somewhat exaggerated the hypoxic and inactivity/unloading conditions, that are expected in the Lunar habitats. Nevertheless, our results clearly demonstrate that combining BR and hypoxia detrimentally affects psychological responses, despite a large inter- and intra-individual variability. Research findings emphasize the importance of cognitive function, psychological responses, and social interactions between crewmembers to ensure a successful space mission (Palinkas, 2001; Manzey, 2004; Kanas et al., 2009). Individuals' psychological profile, personality and cognitive characteristics, as well as, interpersonal and psychosocial issues are closely linked to astronauts' behavior and performance.

### Implications for hypoxic/altitude training

Hypoxic training is becoming increasingly popular in competitive sports. Athletes live and train at altitudes, to take advantage of the altitude-related hematological and non-hematological adaptations. Some others prepare for competitions at altitude and sea level, by living in hypoxic conditions and training at low altitudes or also employ different intermittent hypoxic protocols (Millet et al., 2010). Regardless of which training regimen is adopted, the focus to date has been on the benefits of such training in terms of increased physiological indices, such as erythropoiesis and the subsequent increase in erythrocytes and hemoglobin. The deleterious effects that hypoxia may have on athletes have not been equally scrutinized. The hypophagia and hypodipsia associated with altitude exposure will certainly affect the nutritional and hydration state of the athlete and may consequently affect performance. Similarly, hypoxia-induced central sleep apnoea will affect the sleep architecture and may also have a negative influence on performance. The present study demonstrates that hypoxia increases the negative psychological responses in conditions of extreme physical inactivity. On the other hand, the results demonstrate that the observed hypoxic effect can be counteracted by daily physical activity. However, the activity levels in the present study were designed to mimic the activity during the participants' normal daily activity. Whether hypoxic exposure in combination with strenuous exercise training has a similar effect on psychological responses requires further investigation.

### Study limitations and methodological considerations

While the present study provides an important insight into the independent and combined effects of hypoxia and inactivity on psychological status, there are certain limitations that should be taken into account when interpreting the obtained outcomes. Firstly, although we focused on the analysis of the group/condition average values a significant inter- and intra-individual variability was noted during all hypoxic and/or bed rest exposures indicating that this approach might be limited to effectively and comprehensively capture participants' psychological responses. Secondly, participants' personality characteristics should be taken into account by future prospective studies as they constitute an important variable that can modify participants' mental and cognitive ability in successfully adapting to adverse environments. Finally, due to the project's logistical and financial constraints the sample size employed in this study is limited and should be considered when interpreting the results. Indeed, this limitation is common to all such bed rest studies. Although difficult, prospective projects in this area should aim for larger sample sizes that would enable improved and more accurate estimations of the psychological changes induced by various environmental factors. Based on the above, future, controlled studies are clearly needed and should aim to account for participants' personality characteristics and also consider significant between-individual variability in responses to the tested stressors (i.e., confinement, (in)activity, and hypoxia).

## Conclusion

We conclude that hypoxia enhances the negative psychological characteristics in inactive, bed ridden participants, but not in active, ambulatory participants. In line with our previous results (Stavrou et al., 2015), the present outcomes further demonstrate that the hypoxic effect on mood is transient, is only present during the hypoxic stimulus; and is not sustained once the hypoxic stimulus is removed, nor does it appear to be dependent on the duration of the hypoxic inactivity. Our results also suggest that a small volume of daily physical activity is sufficient to negate the negative emotional responses induced by a hypoxic environment. Thus, it seems warranted to further explore the effect of exercise as a means of improving the psychological responses during prolonged hypoxic/altitude exposures, for space settings as well as for sport/clinical applications. Finally, the present study also provides an impetus for further investigations into the manner by which increased activity may improve the quality of life of patients rendered hypoxic and inactive by various clinical conditions.

## Author contributions

IM and OE initiated and designed the study. Together with TD and NS they performed the measurements, analysis, and interpretation of the data. All authors participated in the preparation of the manuscript.

### Conflict of interest statement

The authors declare that the research was conducted in the absence of any commercial or financial relationships that could be construed as a potential conflict of interest. The handling editor declared a shared affiliation, though no other collaboration, with one of the authors IBM.

## References

[B1] Acevedo E. O. Ekkekakis P. (2001). The transactional psychobiological nature of cognitive appraisal during exercise in environmentally stressful conditions. Psychol. Sport Exerc. 2, 47–67. 10.1016/S1469-0292(00)00013-3

[B2] Bahrke M. S. Shukitt-Hale B. (1993). Effects of altitude on mood, behaviour and cognitive functioning: a review. Sports Med. 16, 97–115. 10.2165/00007256-199316020-00003 8378672

[B3] Banderet L. E. Burse R. L. (1991). Effects of high terrestrial altitude on military performance, in Handbook of Military Psychology, eds Gal R. Mangelsdorff A. D. (New York, CA: John Wiley & Sons), 233–254.

[B4] Barbour K. A. Blumenthal J. A. (2005). Exercise training and depression in older adults. Neurobiol. Aging 26, 119–123. 10.1016/j.neurobiolaging.2005.09.007 16223547

[B5] Bärtsch P. Saltin B. (2008). General introduction to altitude adaptation and mountain sickness. Scand. J. Med. Sci. Sports 1(Suppl.), 1–10. 10.1111/j.1600-0838.2008.00827.x 18665947

[B6] Cronbach L. J. (1951). Coefficient alpha and the internal structure of tests. Psychometrika 16, 297–334. 10.1007/BF02310555

[B7] Curran S. L. Andrykowski M. A. Studts J. L. (1995). Short form of the Profile of Mood States (POMS-SF): psychometric information. Psychol. Assess. 7, 80–83. 10.1037/1040-3590.7.1.80

[B8] Davidson R. J. (2001). The neural circuitry of emotion and affective style: prefrontal cortex and amygdala contributions. Soc. Sci. Inf. 40, 11–37. 10.1177/053901801040001002

[B9] Debevec T. Bali T. C. Simpson E. J. MacDonald I. A. Eiken O. Mekjavic I. B. (2014). Separate and combined effects of 21-day bed rest and hypoxic confinement on body composition. Eur. J. Appl. Physiol. 114, 2411–2425. 10.1007/s00421-014-2963-1 25091855

[B10] De La Torre G. G. van Baarsen B. Ferlazzo F. Kanas N. Weiss K. Scheider S. (2012). Future perspectives on space psychology: recommendations on psychosocial and neurobehavioural aspects on human spaceflight. Acta Astronaut. 81, 587–599. 10.1016/j.actaastro.2012.08.013

[B11] DeRoshia C. W. Greenleaf J. E. (1993). Performance and mood-state parameters during 30-day 6° head-down bed rest with exercise training. Aviat. Space Environ. Med. 64, 522–527. 8338499

[B12] Edwards H. Rose E. A. Schorow M. King T. C. (1981). Postoperative deterioration in psychomotor function. J. Am. Med. Assoc. 245, 1342–1343. 10.1001/jama.1981.03310380046026 6110788

[B13] Eiken O. Mekjavic I. B. (2015). Proceedings of the ESA Topical Team Workshop on simulation of Lunar Habitats, in Book of Abstracts of the 36th Annual Meeting of the International Society for Gravitational Physiology, ed Mekjavic I. B. (Ljubljana: Sudio Print do.o.o.), 133–139.

[B14] Ekkekakis P. (2003). Pleasure and displeasure from the body: perspective from exercise. Cogn. Emot. 17, 213–239. 10.1080/02699930302292 29715726

[B15] Ekkekakis P. Lind E. (2006). Exercise does not feel the same when you are overweight: the impact of self-selected and imposed intensity on affect and exertion. Int. J. Obes. 30, 652–660. 10.1038/sj.ijo.0803052 16130028

[B16] Erickson K. I. Hillman C. H. Kramer A. F. (2015). Physical activity, brain, and cognition. Curr. Opin. Behav. Sci. 4, 27–32. 10.1016/j.cobeha.2015.01.005

[B17] Field A. (2009). Discovering Statistics Using SPSS. Thousand Oaks, CA: SAGE Publications.

[B18] Gallagher S. A. Hackett P. H. (2004). High-altitude illness. Emerg. Med. Clin. North Am. 22, 329–355. 10.1016/j.emc.2004.02.001 15163571

[B19] Gaul C. Schmidt T. Windisch G. Wieser T. Müller T. Vielhaber S.. (2006). Subtle cognitive dysfunction in adult onset myotonic dystrophy type I (DM1) and type 2 (DM2). Neurology 67, 350–352. 10.1212/01.wnl.0000225180.27833.c1 16864839

[B20] Gordon B. A. Rykhlevskaia E. I. Brumback C. R. Lee Y. Elavsky S. Konopack J. F.. (2008). Neuroanatomical correlates of aging, cardiopulmonary fitness level, and education. Psychophysiology 45, 825–838. 10.1111/j.1469-8986.2008.00676.x 18627534 PMC5287394

[B21] Hillman C. H. Erickson K. I. Kramer A. F. (2008). Be smart, exercise your heart: exercise effects on brain and cognition. Nat. Rev. Neurosci. 9, 58–65. 10.1038/nrn2298 18094706

[B22] Hornbein T. F. (2001). The high-altitude brain. J. Exp. Biol. 204, 3129–3132. 11581326 10.1242/jeb.204.18.3129

[B23] Hornbein T. F. Townes B. D. Schoene R. B. Sutton J. R. Houston C. S. (1989). The cost to the central nervous system of climbing to extremely high altitude. N. Engl. J. Med. 321, 1714–1719. 10.1056/NEJM198912213212505 2512483

[B24] Hötting K. Röder B. (2013). Beneficial effects of physical exercise on neuroplasticity and cognition. Neurosci. Biobehav. Rev. 37, 2243–2257. 10.1016/j.neubiorev.2013.04.005 23623982

[B25] Ishizaki Y. Ishizaki T. Fukuoka H. Kim C.-S. Fujita M. Maegawa Y.. (2002). Changes in mood status and neurotic levels during a 20-day bed rest. Acta Astronaut. 50, 453–459. 10.1016/S0094-5765(01)00189-8 11924678

[B26] Kanas N. A. Salnitskiy V. P. Ritsher J. B. Gushin V. I. Weiss D. S. Saylor S. A. (2007). Psychosocial interactions during ISS missions. Acta Astronaut. 60, 329–335. 10.1016/j.actaastro.2006.09.001

[B27] Kanas N. Sandal G. Boyd J. E. Gushin V. I. Manzey D. North R. (2009). Psychology and culture during long-duration space missions. Acta Astronaut. 64, 659–677. 10.1016/j.actaastro.2008.12.005

[B28] Lind E. Joehns-Matre R. R. Ekkekakis P. (2005). What intensity of physical activity do previously sedentary middle-aged women select? Evidence of a coherent pattern from physiological, perceptual and affective markers. Prev. Med. 40, 407–419. 10.1016/j.ypmed.2004.07.006 15530593

[B29] Lipnicki D. M. Gunga H.-C. (2009). Physical inactivity and cognitive functioning: results from bed-rest studies. Eur. J. Appl. Physiol. 105, 27–35. 10.1007/s00421-008-0869-5 18797919

[B30] Liu Q. Zhou R. Chen S. Tan C. (2012). Effects of head-down bed rest on the executive functions and emotional response. PLOS ONE 7:e52160. 10.1371/journal.pone.0052160 23284916 PMC3524097

[B31] Manzey D. (2004). Human missions to Mars: new psychological challenges and research issues. Acta Astronaut. 55, 781–790. 10.1016/j.actaastro.2004.05.013 15806750

[B32] McDonald C. M. (2002). Physical activity, health impairments, and disability in neuromuscular disease. Am. J. Phys. Med. Rehab. 81, S108–S120. 10.1097/00002060-200211001-00012 12409816

[B33] Mikkelsen R. L. Middelboe T. Pisinger C. Stage K. B. (2004). Anxiety and depression in patients with chronic obstructive pulmonary disease (COPD): a review. Nord. J. Psychiatry 58, 65–70. 10.1080/08039480310000824 14985157

[B34] Millet G. P. Roels B. Schmitt L. Woorons X. Richalet J. P. (2010). Combining hypoxic methods for peak performance. Sports Med. 40, 1–25. 10.2165/11317920-000000000-00000 20020784

[B35] Palinkas L. A. (2001). Psychological issues in long-term space flight: Overview. Gravit. Space Bio. Bull. 14, 25–33. 11865866

[B36] Park D. C. Glass J. M. Minear M. Crofford L. J. (2001). Cognitive function in fibromyalgia patients. Arthritis Rheum. 44, 2125–2133. 10.1002/1529-0131(200109)44:9<2125::AID-ART365>3.0.CO;2-1 11592377

[B37] Pavy-Le Traon A. Heer M. Narici M. V. Rittweger J. Vernikos J. (2007). From space to Earth: advances in human physiology from 20 years of bed rest studies (1986-2006). Eur. J. Appl. Physiol. 101, 143–194. 10.1007/s00421-007-0474-z 17661073

[B38] Ritz T. Steptoe A. DeWilde S. Costa M. (2000). Emotions and stress increase respiratory resistance in asthma. Psychosom. Med. 62, 401–412. 10.1097/00006842-200005000-00014 10845354

[B39] Roach R. Bartsch P. Hackett P. Oelz O. (1993). The Lake Louse AMS Scoring Concensus Committee. The Lake Louise acute mountain sickness scoring system, in Hypoxia and Molecular Medicine, eds Sutton J. Houston C. Coates G. (Burlington, VT: Queen City Printers), 272–274.

[B40] Russo-Neustadt A. A. Chen M. J. (2005). Brain-derived neurotrophic factor and antidepressant activity. Curr. Pharm. Des. 11, 1495–1510. 10.2174/1381612053764788 15892658

[B41] Ryback R. S. Trimble R. W. Lewis O. F. Jennings C. L. (1971). Psychobiological effects of prolonged weight-lessness (bed rest) in young health volunteers. Aerosp. Med. 42, 408–415. 4343907

[B42] Shacham S. (1983). A shortened version of the Profile of Mood States. J. Pers. Assess. 47, 305–306. 10.1207/s15327752jpa4703_14 6886962

[B43] Shehab R. L. Schlegel R. E. Schiflett S. G. Eddy D. (1998). The NASA performance assessment workstation: cognitive performance during head-down bed rest. Acta Astronaut. 43, 223–233. 10.1016/S0094-5765(98)00156-8 11541926

[B44] Stavrou N. A. McDonnell A. C. Eiken O. Mekjavic I. B. (2015). Psychological strain: examining the effect of hypoxic bedrest and confinement. Physiol. Behav. 139, 497–504. 10.1016/j.physbeh.2014.12.015 25484354

[B45] Tabachnick B. G. Fidell L. S. (2006). Using Multivariate Statistics. Boston, MA: Pearson.

[B46] Troosters T. van der Molen T. Polkey M. Rabinovich R. A. Vogiatzis I. (2013). Improving physical activity in COPD: towards a new paradigm. Respir. Res. 14:115. 10.1186/1465-9921-14-115 24229341 PMC4176094

[B47] Van Baarsen B. Ferlazzo F. Ferravante D. DiNocera F. Jörgensen J. Smit J. H. (2009). Digging into space psychology and isolation: the MARS520 LODGEAD study: Preliminary results of the MARS105 pilot study, in Proceedings of the 60th International Astronautical Congress (Daejeon).

[B48] Virués-Ortega J. Buela-Casal G. Garrido E. Alcázar B. (2004). Neuropsychological functioning associated with high-altitude exposure. Neuropsychol. Rev. 14, 197–224. 10.1007/s11065-004-8159-4 15796116

[B49] Watson D. Clark L. A. Tellegen A. (1988). Development and validation of brief measures of positive and negative affect: the PANAS Scales. J. Pers. Soc. Psychol. 47, 1063–1070. 10.1037/0022-3514.54.6.1063 3397865

[B50] Weinstein A. M. Voss M. W. Prahash R. S. Chaddock L. Szabo A. White S. M.. (2012). The association between aerobic fitness and executive function is mediated by prefrontal cortex volume. Brain Behav. Immun. 26, 811–819. 10.1016/j.bbi.2011.11.008 22172477 PMC3321393

[B51] Wilson I. (2006). Depression in the patient with COPD. Int. J. Chron. Obstruct. Pulmon. Dis. 1, 61–64. 10.2147/copd.2006.1.1.61 18046903 PMC2706604

[B52] Winget C. M. DeRoshia C. W. (1986). Psychosocial and chronophysiological effects of inactivity and immobilizatio, in Inactivity: Physiological Effects, eds Sandler H. Vernikos J. (New York, CA: Academic Press), 123–147.

[B53] Yeung R. R. (1996). The acute effects of exercise on mood state. J. Psychosom. Res. 40, 123–141. 10.1016/0022-3999(95)00554-4 8778396

